# Lipoprotein combined index and prevalent hyperuricemia among normolipidemic oilfield workers: a cross-sectional study

**DOI:** 10.3389/fendo.2026.1768222

**Published:** 2026-03-26

**Authors:** Haobiao Liu, Jing Tang, Yingjie Cai, Licheng Yang, Qingsong Li, Xuefeng Yu, Husna Wali, Abebe Feyissa Amhare, Zhiyong Du, Ziwei Guo, Jing Han, Tao Zhu

**Affiliations:** 1 Xi’an Gem Flower Changqing Hospital, Xi’an, Shaanxi, China; 2 School of Public Health, Health Science Center, Xi’an Jiaotong University, Xi’an, Shaanxi, China; 3 Department of Comprehensive Orthopedics, The Second Affiliated Hospital of Xi’an Jiaotong University, Xi’an, Shaanxi, China

**Keywords:** hyperuricemia, lipoprotein combined index, normolipidemia, occupational health, oilfield workers

## Abstract

**Objectives:**

The lipoprotein combined index (LCI) reflects composite lipid metabolic patterns and may capture subtle metabolic disturbances beyond conventional lipid markers. Evidence regarding its relationship with hyperuricemia among normolipidemic individuals is scarce. This study investigated the association between LCI and hyperuricemia in an occupational population with normal lipid levels.

**Methods:**

A total of 2,029 normolipidemic oilfield workers were included. Hyperuricemia was primarily defined using sex-specific serum uric acid thresholds. Associations between LCI and hyperuricemia were examined using logistic regression, with dose-response relationships explored by restricted cubic spline (RCS) analysis. Prespecified subgroup and multiple sensitivity analyses were conducted to assess robustness.

**Results:**

Hyperuricemia prevalence rose from 9.25% in the lowest LCI quartile to 28.99% in the highest. In fully adjusted models, each standard deviation increase in LCI was associated with a 37% higher odds of hyperuricemia (OR = 1.37, 95% CI: 1.20–1.58, *P* < 0.001). Compared with the lowest quartile, participants in the highest quartile had more than double the odds (OR = 2.18, 95% CI: 1.45–3.30, *P* < 0.001), with a significant linear trend (*P* for trend < 0.001). RCS analysis further supported a positive and linear association between LCI and prevalent hyperuricemia (*P* for nonlinear = 0.427). Subgroup analyses showed consistent positive associations across all strata, with numerically higher ORs observed in older and female individuals, and no statistically significant interactions were detected (all *P* for interaction > 0.05). All sensitivity analyses yielded similar results.

**Conclusions:**

Higher LCI levels were associated with increased odds of hyperuricemia among normolipidemic oilfield workers. LCI may serve as an early marker of urate-related metabolic dysregulation, offering additional value beyond traditional lipid indicators. Longitudinal studies are warranted to confirm its predictive potential.

## 1 Introduction

Hyperuricemia, defined as elevated serum uric acid beyond physiological thresholds, is increasingly recognized not only as a precursor to gout but also as an independent risk factor for a broad array of metabolic and endocrine disorders (1, 2). Recent epidemiological studies have documented the growing prevalence of hyperuricemia worldwide (3–5), highlighting the necessity to elucidate its underlying determinants beyond classical risk factors.

Dyslipidemia, characterized by abnormalities in traditional lipid parameters such as total cholesterol (TC), triglycerides (TG), low-density lipoprotein cholesterol (LDL-C), and high-density lipoprotein cholesterol (HDL-C), has long been implicated in metabolic derangements. Multiple cross-sectional studies have reported positive associations between traditional lipid parameters and hyperuricemia (2, 6). Importantly, prospective longitudinal evidence also supports this relationship. In a six-year Chinese cohort, individuals with elevated TG and lower HDL-C had a significantly higher incidence of hyperuricemia over time, indicating a temporal association beyond cross-sectional correlations (7). Similarly, a large health examination cohort showed that changes in TG and HDL-C over follow-up predicted future development of hyperuricemia (8).

However, reliance on individual lipid parameters may inadequately reflect the complex interplay of lipoprotein fractions and their metabolic impact. Recently, composite lipid indices have been proposed as more integrated markers of atherogenic and metabolic risk. One such index, the lipoprotein combined index (LCI), calculated from standard lipid panel components, has shown promise (9). Unlike single lipid measures, LCI integrates multiple atherogenic lipid fractions relative to anti-atherogenic lipoprotein fractions, thereby reflecting the overall balance between pro-atherogenic and protective lipoproteins and potentially capturing subtle lipid metabolic disturbances not apparent from individual lipid parameters alone. For example, a large longitudinal study among non-obese, normolipidemic Chinese individuals demonstrated that higher LCI at baseline was independently associated with elevated risk of developing non-alcoholic fatty liver disease (NAFLD) over five years, even when traditional lipid parameters were within normal ranges (10). In that study, LCI outperformed individual lipid measures in predicting non-alcoholic fatty liver disease risk, suggesting its higher sensitivity for detecting subclinical lipid metabolic disturbances. Beyond liver disease, a growing body of evidence indicates that composite atherogenic lipid indices, such as the non–HDL-C to HDL-C ratio (NHHR) and TG/HDL-C ratio, are associated with hyperuricemia across different populations (11, 12). Collectively, these indices capture lipid metabolic imbalance characterized by an excess of atherogenic lipoproteins relative to protective fractions. In this context, an elevated LCI may similarly reflect a more pronounced and integrative disturbance in lipid metabolism, potentially linking dyslipidemic patterns to urate dysregulation more sensitively than single lipid parameters.

Nevertheless, to our knowledge, no study to date has examined the association between LCI and hyperuricemia, particularly among individuals with ostensibly normal lipid profiles. This gap is especially relevant in occupational populations, such as oilfield workers, who may present unique metabolic risk profiles due to disruption of circadian rhythm, irregular meal schedules, and high physical demands that may interact with lipid and purine metabolism in complex ways. Yet, few epidemiological studies have targeted such populations to explore lipid–uric acid interactions under normolipidemic conditions.

Accordingly, we conducted a cross-sectional study among normolipidemic oilfield workers to examine whether elevated LCI is associated with increased odds of hyperuricemia. We hypothesize that even within a population with normal standard lipid profiles, higher LCI will be associated with hyperuricemia. Our study seeks to contribute novel evidence on the role of subclinical lipid metabolic dysregulation—as captured by LCI—in uric acid metabolism and metabolic risk, thereby identifying high-risk people in oilfield environments earlier and directing more focused preventative measures to lessen the growing prevalence of hyperuricemia.

## 2 Materials and methods

### 2.1 Study design and population

This cross-sectional study was conducted among oilfield workers who underwent routine occupational health examinations at a hospital in Xi’an, China, between October and December 2022. During the health examination, workers were invited to participate in an additional questionnaire survey for research purposes. Details of the data collection procedures, health examination protocols, and questionnaire administration have been described previously (13). Briefly, the examination incorporated standardized clinical measurements, laboratory testing, and structured interviews administered by trained personnel. Among workers attending the routine health examination during the study period, those who completed both the questionnaire survey and the required laboratory assessments were considered for inclusion.

**Figure 1:**
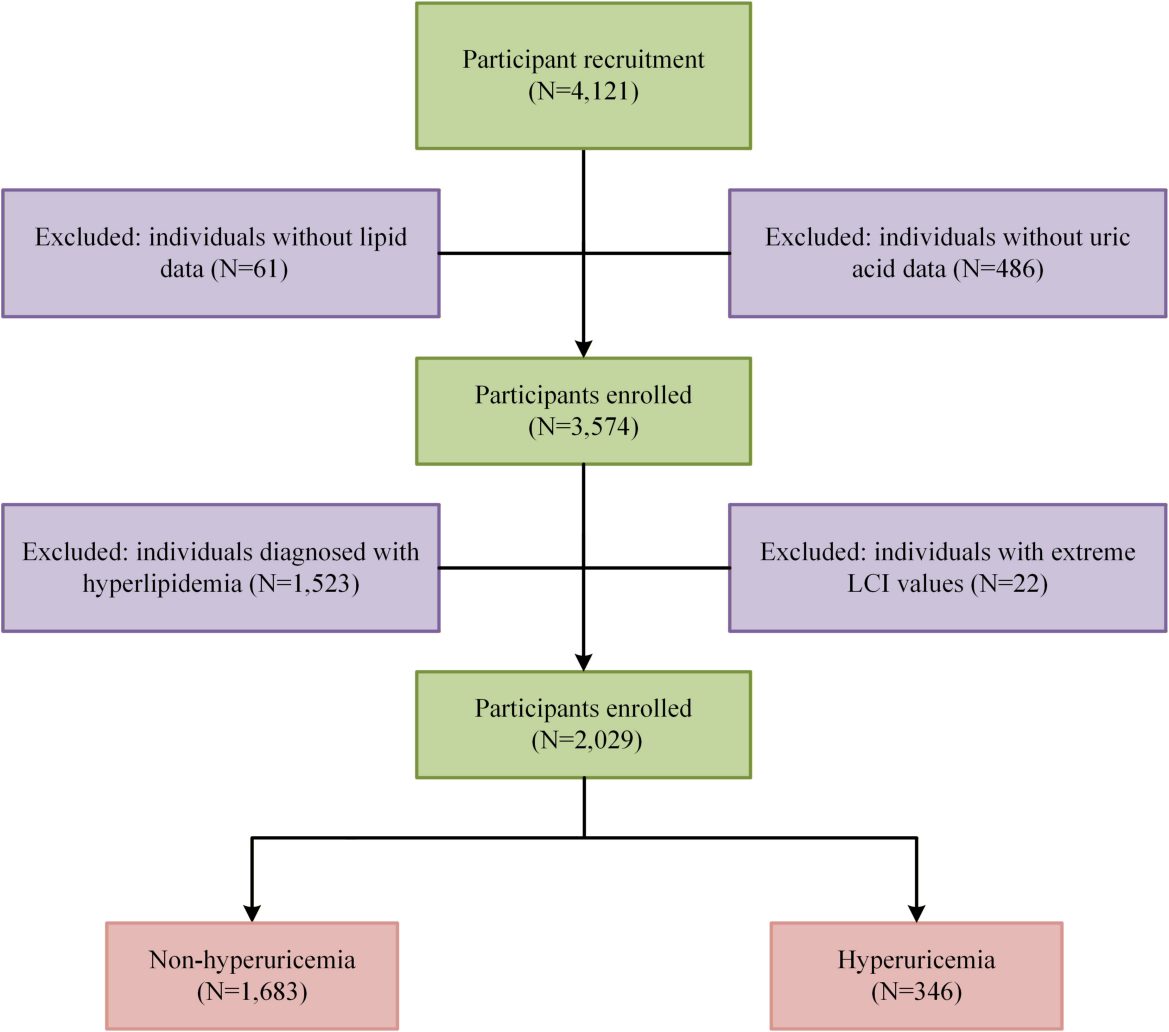
Flow diagram of participants included in this study.

A total of 4,121 individuals were initially considered for eligibility. Participants were excluded if they had missing serum uric acid measurements (N = 486), missing lipid profile data (N = 61), hyperlipidemia defined as lipid concentrations exceeding guideline-recommended thresholds at the health examination or a self-reported physician diagnosis or use of lipid-lowering medication (N = 1,523), or extreme LCI values exceeding three standard deviations (SD) from the mean to minimize the influence of extreme outliers on model stability, a commonly adopted approach in epidemiological analyses (14, 15) (N = 22). Information on workers who attended the health examination but did not complete the questionnaire was not available for analysis. After these exclusions, 2,029 normolipidemic workers were included in the final analysis (**Figure 1**).

### 2.2 Assessment of exposure and outcome

Fasting venous blood samples were collected in the morning after an overnight fast. Serum concentrations of total cholesterol, triglycerides, high-density lipoprotein cholesterol, and low-density lipoprotein cholesterol were measured using an automated biochemical analyzer in the hospital’s central laboratory following standard quality-control procedures. The LCI was then calculated as: LCI = (TC × TG × LDL-C)/HDL-C. In the study population, LCI values ranged from 0.75 to 35.58, with a mean of 11.61. In the primary analyses, LCI was examined both as a continuous and a categorical variable. For continuous analyses, LCI values were standardized using a z-score transformation to improve comparability and model convergence. For categorical analyses, participants were classified into quartiles according to the distribution of LCI, with cutoff points at the 25th (5.86), 50th (9.94), and 75th (15.99) percentiles.

Serum uric acid concentrations were measured using the uricase–peroxidase enzymatic colorimetric method on an automated biochemical analyzer. Hyperuricemia was defined using sex-specific thresholds, with serum uric acid levels greater than 420 μmol/L (7.0 mg/dL) in male participants and greater than 360 μmol/L (6.0 mg/dL) in female participants (16).

Hyperlipidemia is defined as TC ≥ 6.2 mmol/L, TG ≥ 2.3 mmol/L, LDL-C ≥ 4.1 mmol/L, HDL-C < 1.0 mmol/L, self-reported physician diagnosis of hyperlipidemia, or current use of lipid-lowering medication.

### 2.3 Covariates

Covariates were selected based on prior literature and biological relevance (13). Demographic factors included age, sex, ethnicity, education level, marital status, and annual income. Occupational variables included shift work, chemical substance exposure, noise exposure, and dust exposure. Lifestyle characteristics included cigarette smoking, alcohol drinking, tea drinking, physical activity, salt intake, and food diversity. Clinical factors included body mass index (BMI), estimated glomerular filtration rate (eGFR), hypertension, diabetes, and cardiovascular disease. Detailed definitions of covariates are presented in **Supplementary Table 1**.

### 2.4 Statistical analysis

Descriptive statistics were computed for all variables. Continuous variables were summarized as mean ± SD, and categorical variables as numbers and percentages. Differences across LCI quartiles were examined using analysis of variance or chi-square tests. As several covariates contained missing values, with missing rates for all variables below 12% (**Supplementary Table 2**), multiple imputation by chained equations was performed with five imputations. The imputation models included all covariates used in the multivariable analyses, while exposure and outcome variables were complete and therefore not imputed. Convergence and plausibility of the imputation models were assessed using standard diagnostic procedures, and estimates were combined across imputed datasets using Rubin’s rules.

Multivariable logistic regression was used to evaluate the association between LCI and hyperuricemia, with results presented as odds ratios (ORs) and 95% confidence intervals (CIs). LCI was examined both as a standardized continuous variable (per SD increase) and as a categorical variable based on quartiles. Linear trend tests were performed by assigning the median value of each quartile and modeling it as a continuous variable. Three hierarchical models were constructed to progressively control for potential confounders. Model 1 included no covariates. Model 2 adjusted for demographic factors. Model 3 further accounted for occupational variables, lifestyle characteristics, and clinical factors. To explore the potential dose-response relationship between LCI and hyperuricemia, restricted cubic spline (RCS) analysis was fitted using three knots placed at the 10th, 50th, and 90th percentiles of LCI (17).

Potential effect modification was evaluated across predefined subgroups, including age (<40 vs. ≥40 years), sex (male vs. female), shift schedule (no vs. yes), and BMI (underweight/normal vs. overweight/obesity). To assess robustness, several sensitivity analyses were conducted, including a complete-case analysis excluding individuals with any missing covariate data, applying the Chinese clinical practice guideline definition of hyperuricemia (serum uric acid greater than 420 μmol/L for both sexes), repeating the primary regression analyses after re-including participants with extreme LCI values to evaluate the potential influence of outliers, and categorizing LCI into tertiles.

All statistical analyses were performed using R software (version 4.4.0). A two-sided *P* value <0.05 was considered statistically significant.

## 3 Result

### 3.1 Baseline characteristics of participants

A total of 2,029 normolipidemic oilfield workers were included in the final analysis. **Table 1** presents the participants’ characteristics across quartiles of the LCI. Several demographic and lifestyle factors differed significantly across LCI categories.

**Table 1:** Baseline characteristics of participants based on lipoprotein combined index quartiles.

Variables	Lipoprotein combined index					*P* value
	Total (N = 2,029)	Quartile 1 (N = 508)	Quartile 2 (N = 507)	Quartile 3 (N = 507)	Quartile 4 (N = 507)	
Age, year	40.98 (8.32)	38.81 (7.93)	41.25 (8.41)	41.60 (8.32)	42.24 (8.21)	<0.001
Sex						<0.001
Male	1088 (53.62)	176 (34.65)	228 (44.97)	308 (60.75)	376 (74.16)	
Female	941 (46.38)	332 (65.35)	279 (55.03)	199 (39.25)	131 (25.84)	
Ethnicity						0.895
Han	1989 (98.03)	497 (97.83)	497 (98.03)	499 (98.42)	496 (97.83)	
Other	40 (1.97)	11 (2.17)	10 (1.97)	8 (1.58)	11 (2.17)	
Education level						0.113
High school or below	723 (35.63)	157 (30.91)	177 (34.91)	200 (39.45)	189 (37.28)	
College degree	760 (37.46)	200 (39.37)	191 (37.67)	175 (34.52)	194 (38.26)	
University graduate or above	546 (26.91)	151 (29.72)	139 (27.42)	132 (26.04)	124 (24.46)	
Marital status						0.018
Married	1714 (84.48)	407 (80.12)	439 (86.59)	434 (85.60)	434 (85.60)	
Unmarried/Separated	315 (15.52)	101 (19.88)	68 (13.41)	73 (14.40)	73 (14.40)	
Annual income, thousand (CNY)						0.545
≤ 100	440 (21.69)	118 (23.23)	108 (21.30)	107 (21.10)	107 (21.10)	
101-150	1227 (60.47)	308 (60.63)	317 (62.52)	305 (60.16)	297 (58.58)	
≥ 151	362 (17.84)	82 (16.14)	82 (16.17)	95 (18.74)	103 (20.32)	
Shift work						0.024
No	588 (28.98)	123 (24.21)	144 (28.40)	157 (30.97)	164 (32.35)	
Yes	1441 (71.02)	385 (75.79)	363 (71.60)	350 (69.03)	343 (67.65)	
Chemical substance exposure						0.595
No	431 (21.24)	108 (21.26)	103 (20.32)	118 (23.27)	102 (20.12)	
Yes	1598 (78.76)	400 (78.74)	404 (79.68)	389 (76.73)	405 (79.88)	
Noise exposure						0.150
No	747 (36.82)	181 (35.63)	182 (35.90)	208 (41.03)	176 (34.71)	
Yes	1282 (63.18)	327 (64.37)	325 (64.10)	299 (58.97)	331 (65.29)	
Dust exposure						0.687
No	1494 (73.63)	384 (75.59)	371 (73.18)	372 (73.37)	367 (72.39)	
Yes	535 (26.37)	124 (24.41)	136 (26.82)	135 (26.63)	140 (27.61)	
Cigarette smoking						<0.001
No	1287 (63.43)	396 (77.95)	349 (68.84)	293 (57.79)	249 (49.11)	
Yes	742 (36.57)	112 (22.05)	158 (31.16)	214 (42.21)	258 (50.89)	
Alcohol drinking						<0.001
No	1402 (69.10)	411 (80.91)	365 (71.99)	330 (65.09)	296 (58.38)	
Yes	627 (30.90)	97 (19.09)	142 (28.01)	177 (34.91)	211 (41.62)	
Tea drinking						<0.001
No	1050 (51.75)	305 (60.04)	281 (55.42)	243 (47.93)	221 (43.59)	
Yes	979 (48.25)	203 (39.96)	226 (44.58)	264 (52.07)	286 (56.41)	
Physical activity						0.055
Inactive	467 (23.02)	96 (18.90)	130 (25.64)	125 (24.65)	116 (22.88)	
Active	1562 (76.98)	412 (81.10)	377 (74.36)	382 (75.35)	391 (77.12)	
Salt intake						0.026
≤ 6 grams/day	1041 (51.31)	276 (54.33)	278 (54.83)	238 (46.94)	249 (49.11)	
> 6 grams/day	988 (48.69)	232 (45.67)	229 (45.17)	269 (53.06)	258 (50.89)	
Food diversity						0.760
< 4 types/day	942 (46.43)	226 (44.49)	242 (47.73)	238 (46.94)	236 (46.55)	
≥ 4 types/day	1087 (53.57)	282 (55.51)	265 (52.27)	269 (53.06)	271 (53.45)	
Body mass index, Kg/m^2^						<0.001
Underweight/Normal	1250 (61.61)	423 (83.27)	348 (68.64)	281 (55.42)	198 (39.05)	
Overweight	593 (29.23)	69 (13.58)	119 (23.47)	179 (35.31)	226 (44.58)	
Obesity	186 (9.17)	16 (3.15)	40 (7.89)	47 (9.27)	83 (16.37)	
eGFR, mL/min/1.73 m^2^	110.41 (10.27)	113.19 (10.31)	110.53 (10.22)	109.46 (9.44)	108.47 (10.48)	<0.001
Hypertension						<0.001
No	1698 (83.69)	457 (89.96)	430 (84.81)	427 (84.22)	384 (75.74)	
Yes	331 (16.31)	51 (10.04)	77 (15.19)	80 (15.78)	123 (24.26)	
Diabetes						0.001
No	1938 (95.52)	493 (97.05)	486 (95.86)	491 (96.84)	468 (92.31)	
Yes	91 (4.48)	15 (2.95)	21 (4.14)	16 (3.16)	39 (7.69)	
Cardiovascular disease						0.006
No	1955 (96.35)	497 (97.83)	494 (97.44)	477 (94.08)	487 (96.06)	
Yes	74 (3.65)	11 (2.17)	13 (2.56)	30 (5.92)	20 (3.94)	
Hyperuricemia						<0.001
No	1683 (82.95)	461 (90.75)	446 (87.97)	416 (82.05)	360 (71.01)	
Yes	346 (17.05)	47 (9.25)	61 (12.03)	91 (17.95)	147 (28.99)	

Participants in the highest LCI quartile tended to be older and predominantly male, with a stepwise increase in age and the proportion of males from Quartile 1 to Quartile 4 (both *P* < 0.001). Marital status and shift work distribution also varied significantly across groups, with workers in higher quartiles more often being married and less likely to engage in shift work. Unhealthy lifestyle patterns were more prevalent among individuals with higher LCI. The proportions of cigarette smoking, alcohol consumption, and tea drinking increased progressively from the lowest to the highest quartile (all *P* < 0.001). Higher LCI levels were also associated with a greater prevalence of overweight and obesity and a modest decline in eGFR (*P* < 0.001). In addition, the prevalence of hypertension, diabetes, and cardiovascular disease increased with rising LCI levels.

Hyperuricemia was most frequent in the highest quartile of LCI (28.99%), compared with 9.25% in Quartile 1 (*P* < 0.001). Baseline characteristics stratified by hyperuricemia status are presented in **Supplementary Table 3**, showing significantly higher LCI values among workers with hyperuricemia. Overall, these findings indicate that workers with elevated LCI values had less favorable metabolic and lifestyle profiles. Correlations between LCI and conventional lipid parameters, as well as selected metabolic indicators, are presented in **Supplementary Figure 1**. LCI showed positive correlations with TC, TG, and LDL-C, and an inverse correlation with HDL-C. In addition, modest positive correlations were observed between LCI and BMI and serum uric acid.

### 3.2 Associations between LCI and hyperuricemia

**Table 2** presents the associations between LCI and hyperuricemia estimated from multivariable logistic regression models. When modeled as a continuous variable, higher LCI was positively associated with the odds of hyperuricemia in all three models. In the unadjusted model (Model 1), higher LCI was strongly associated with hyperuricemia (OR = 1.76, 95% CI: 1.56-1.98, *P* < 0.001). After adjustment for demographic factors in Model 2, the magnitude of the association was modestly reduced but remained statistically significant (OR = 1.63, 95% CI: 1.43-1.85, *P* < 0.001). In the fully adjusted model (Model 3), per one SD increase in LCI was associated with a 37% higher odds of hyperuricemia (OR = 1.37, 95% CI: 1.20-1.58, *P* < 0.001).

**Table 2:** Association between lipoprotein combined index and hyperuricemia among normolipidemic oilfield workers.

Variable	Model 1		Model 2		Model 3	
	OR (95% CI)	*P* value	OR (95% CI)	*P* value	OR (95% CI)	*P* value
Continuous	1.76 (1.56, 1.98)	<0.001	1.63 (1.43, 1.85)	<0.001	1.37 (1.20, 1.58)	<0.001
Categorical						
Quartile 1	1.00 (Reference)		1.00 (Reference)		1.00 (Reference)	
Quartile 2	1.34 (0.90, 2.01)	0.153	1.37 (0.90, 2.10)	0.139	1.10 (0.71, 1.71)	0.659
Quartile 3	2.15 (1.48, 3.15)	<0.001	1.93 (1.30, 2.90)	0.001	1.43 (0.95, 2.18)	0.092
Quartile 4	4.01 (2.82, 5.77)	<0.001	3.41 (2.34, 5.06)	<0.001	2.18 (1.45, 3.30)	<0.001
* P* for trend		<0.001		<0.001		<0.001

Model 1, no covariate was adjusted. Model 2, adjusted for age, sex, ethnicity, education level, marital status, and annual income. Model 3, further adjusted for shift work, chemical substance exposure, noise exposure, dust exposure, cigarette smoking, alcohol drinking, tea drinking, physical activity, salt intake, food diversity, body mass index, estimated glomerular filtration rate, hypertension, diabetes, and cardiovascular disease. OR, odds ratio; CI, confidence interval.

When LCI was analyzed as a categorical variable, higher quartiles were associated with progressively greater odds of hyperuricemia. In the fully adjusted model, compared with individuals in Quartile 1, the odds ratios for hyperuricemia were 1.10 (95% CI: 0.71–1.71) for Quartile 2, 1.43 (95% CI: 0.95–2.18) for Quartile 3, and 2.18 (95% CI: 1.45–3.30) for Quartile 4. Although the associations for Quartiles 2 and 3 were attenuated after multivariable adjustment and did not reach statistical significance, a clear graded relationship remained across increasing LCI categories (*P* for trend < 0.001).

**Figure 2:**
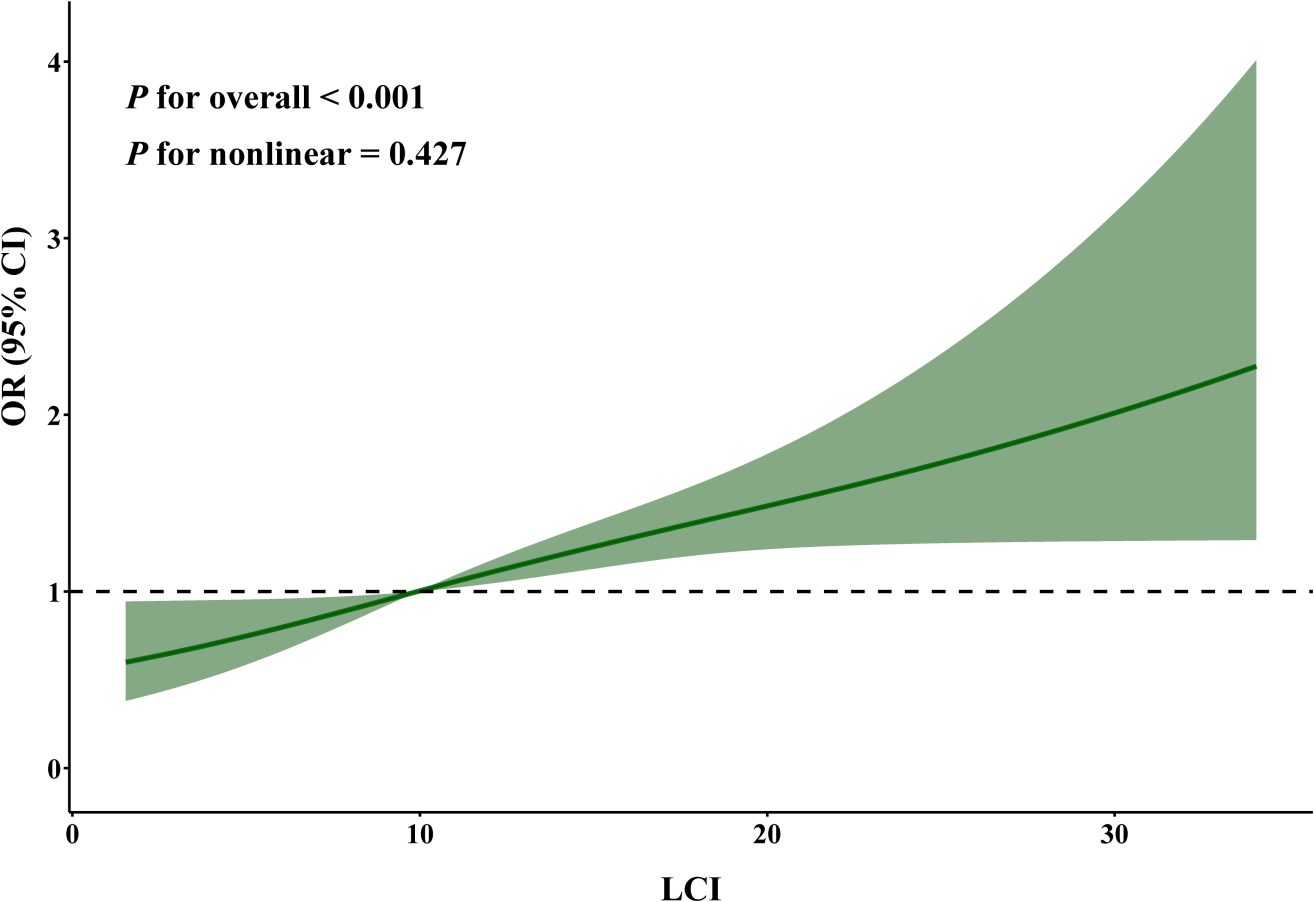
The restricted cubic spline analysis of the association between lipoprotein combined index and hyperuricemia among normolipidemic oilfield workers. The model was adjusted for age, sex, ethnicity, education level, marital status, annual income, shift work, chemical substance exposure, noise exposure, dust exposure, cigarette smoking, alcohol drinking, tea drinking, physical activity, salt intake, food diversity, body mass index, estimated glomerular filtration rate, hypertension, diabetes, and cardiovascular disease. LCI, lipoprotein combined index; OR, odds ratio; CI, confidence interval.

RCS analysis further supported a positive and linear association between LCI and prevalent hyperuricemia (**Figure 2**). The odds of hyperuricemia increased across the observed range of LCI, although estimates at very high LCI values were less precise due to wider confidence intervals (*P* for nonlinear = 0.427).

### 3.3 Subgroup and sensitivity analyses

**Figure 3:**
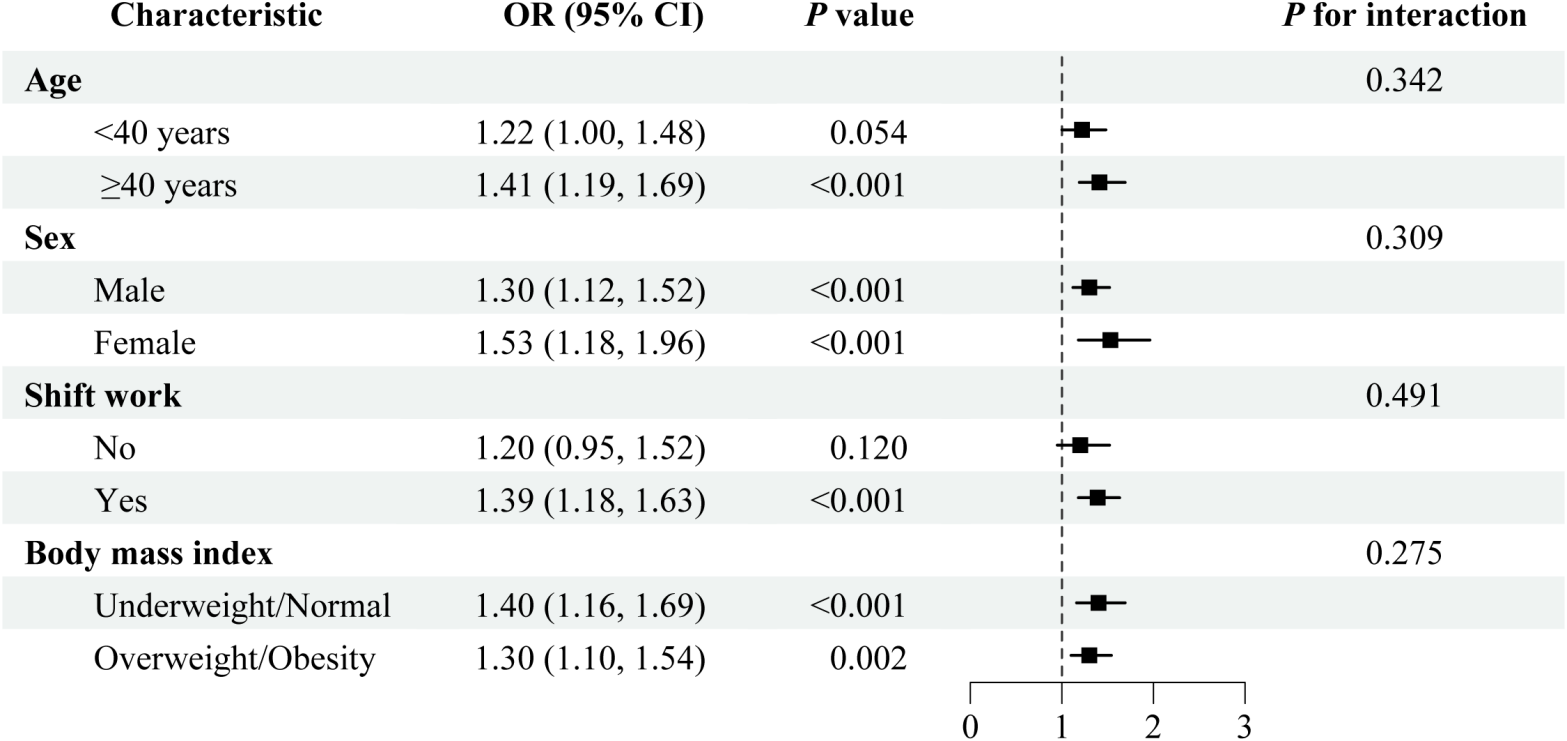
Stratified analyses of the associations between lipoprotein combined index and hyperuricemia among normolipidemic oilfield workers. The model was adjusted for age, sex, ethnicity, education level, marital status, annual income, shift work, chemical substance exposure, noise exposure, dust exposure, cigarette smoking, alcohol drinking, tea drinking, physical activity, salt intake, food diversity, body mass index, estimated glomerular filtration rate, hypertension, diabetes, and cardiovascular disease, except for the corresponding stratification variable. OR, odds ratio; CI, confidence interval.

Subgroup analyses were conducted to explore potential effect modification by age, sex, shift work status, and BMI (**Figure 3**). Overall, the positive association between LCI and hyperuricemia was generally consistent across subgroups, with no statistically significant interactions detected (all *P* for interaction > 0.05), and numerically higher ORs were observed among females and older participants (≥ 40 years). Consistent patterns were observed in subgroup analyses using LCI categorized into quartiles (**Supplementary Table 4**). Across most subgroups, higher LCI quartiles were associated with progressively increased odds of hyperuricemia, indicating a graded relationship. However, similar to the analyses treating LCI as a continuous variable, the associations among participants aged < 40 years and those without shift work were attenuated and not statistically significant.

Sensitivity analyses further supported the robustness of the main findings. Results were largely unchanged after restricting the analysis to participants with complete covariate data, suggesting that missing information did not materially bias the estimates (**Supplementary Table 5**). Applying the Chinese clinical practice guideline definition of hyperuricemia yielded comparable effect sizes and directions, and the association remained statistically significant, although the overall prevalence of hyperuricemia was lower than that observed using sex-specific thresholds (**Supplementary Table 6**). Notably, re-including participants with extreme LCI values did not materially alter the magnitude of the associations (**Supplementary Table 7**). In addition, re-categorizing LCI into tertiles produced results consistent with the primary analyses, with higher LCI categories consistently associated with increased odds of hyperuricemia (**Supplementary Table 8**). Collectively, these sensitivity analyses confirm the stability and reliability of the observed positive association between LCI and hyperuricemia.

## 4 Discussion

In this occupational cross-sectional study, we observed a robust, graded association between the LCI and prevalent hyperuricemia among workers whose conventional lipid panels fell within normal limits. The relationship persisted after comprehensive adjustment for demographic, occupational, lifestyle, and clinical covariates and remained stable across multiple sensitivity analyses. Collectively, these results suggest that lipid-related metabolic risk may already be present at a subclinical stage, even when standard lipid markers appear normal. Accordingly, composite lipid indices such as LCI may uncover subtle lipid abnormalities that are relevant to purine metabolism and urate homeostasis. This observation provides interpretative insight into the metabolic relevance of composite lipid patterns and practical value for identifying individuals with prevalent hyperuricemia in populations traditionally considered metabolically low risk.

Building on this finding, our results extend a substantial body of literature linking traditional lipid parameters to serum uric acid levels. Previous cross-sectional studies conducted across multiple countries have consistently shown that elevated TC, TG, and LDL-C are positively associated with serum uric acid and hyperuricemia, whereas HDL-C tends to show an inverse relationship (2, 18, 19). For instance, a nationally representative sample of U.S. adults demonstrated these associations even after multivariable adjustment (18). Similarly, a large cross-sectional study reported that the TG/HDL-C ratio was positively correlated with serum uric acid and improved hyperuricemia risk stratification beyond individual lipid components (20). Against this background, the present study advances existing evidence by focusing on a novel composite index, LCI, and by restricting the analysis to individuals with normolipidemia. To our knowledge, no previous study has directly examined the association between LCI and hyperuricemia.

Although evidence on LCI and uric acid is lacking, LCI has recently been validated as a sensitive marker for other metabolic disorders (9, 10). Our findings are concordant with these observations and further extend them to uric acid metabolism, suggesting that LCI may function as an integrative and sensitive biomarker for identifying hyperuricemia even when conventional lipid panels fall within reference ranges. Notably, the association between LCI and hyperuricemia was attenuated after further adjustment for cardiometabolic variables in Model 3 compared with Model 2. This attenuation likely reflects adjustment for factors that may lie along the causal pathway linking adverse lipid profiles to hyperuricemia, such as adiposity, impaired renal urate handling, or metabolic comorbidities. Accordingly, Model 3 may represent a more conservative estimate and could partially underestimate the total association. Taken together, these results imply that reliance on standard lipid screening alone may overlook clinically meaningful lipid-related metabolic imbalance relevant to urate dysregulation.

In this regard, the occupational context of oilfield workers warrants particular consideration. This population is characterized by prolonged exposure to complex work-related stressors, including shift work, irregular schedules, physical workload, and potential environmental exposures, which have been linked to disturbances in lipid metabolism, systemic inflammation, oxidative stress, and insulin sensitivity (21, 22). These metabolic perturbations are also closely connected to uric acid production and renal urate handling. As a result, oilfield workers may harbor latent metabolic imbalances that are not readily detected by routine lipid panels, making them a particularly informative population in which to examine the metabolic relevance of composite lipid indices such as LCI.

Importantly, the focus on normolipidemic individuals is not merely semantic but addresses a translationally relevant blind spot in current clinical practice. Standard lipid reference ranges were primarily developed to identify overt atherogenic dyslipidemia and to guide lipid-lowering therapy. However, accumulating evidence indicates that individuals with lipid levels within conventional ranges may still harbor metabolically adverse lipoprotein patterns, such as unfavorable combinations of TG, LDL-C, and low HDL-C, or elevated remnant cholesterol, which confer cardiometabolic and vascular risk beyond individual lipid analytes (9, 23, 24). Large population-based studies have demonstrated that remnant cholesterol is independently associated with atherosclerotic cardiovascular disease and ischemic events, even among individuals with normal LDL-C concentrations (23–25). Moreover, studies employing composite lipid metrics have identified increased risks of metabolic disorders in individuals otherwise classified as metabolically normal based on standard lipid cutoffs (9, 26). From a public health perspective, these findings underscore a potential limitation of single-component lipid screening, as it may provide false reassurance in a substantial subset of individuals. Incorporating an inexpensive calculated index such as LCI could therefore enhance metabolic stratification, helping to identify individuals with prevalent hyperuricemia or adverse metabolic profiles who may warrant closer clinical attention. This consideration is particularly salient in occupational settings, where environmental exposures, shift schedules, and work-related lifestyle patterns may amplify cardiometabolic risk despite normal routine laboratory findings (27, 28). In this context, understanding the metabolic information captured by LCI beyond conventional lipid measures is essential for interpreting its association with hyperuricemia in normolipidemic populations. To further characterize the information captured by LCI, we examined its correlations with conventional lipid components, BMI, and serum uric acid. LCI was strongly correlated with TC, TG, and LDL-C, and inversely correlated with HDL-C, reflecting its composite structure. In contrast, the correlations between LCI and BMI or serum uric acid were of moderate magnitude, suggesting that LCI captures lipid-related metabolic variation that is related to, but not fully explained by, overall adiposity or urate levels alone.

Several pathophysiological mechanisms may plausibly underlie the observed association between LCI and hyperuricemia. These proposed mechanisms are inferred from prior experimental and epidemiological evidence and were not directly assessed in the present study. First, elevated LCI reflects an unfavorable balance between atherogenic lipoproteins and anti-atherogenic HDL, potentially signaling subtle lipoprotein abnormalities not captured by standard lipid measures. Such an imbalance has been linked to altered hepatic lipid handling, impaired insulin sensitivity, and enhanced oxidative stress, all of which are known modulators of uric acid production and renal excretion (29, 30). Second, higher LCI may serve as a surrogate marker for increased remnant lipoproteins or TG-rich lipoproteins, which have been implicated in insulin resistance, metabolic disorders, and renal dysfunction (31–33). Third, accumulation of lipoprotein-derived lipid species within hepatic tissue or the vascular endothelium may promote local inflammation and oxidative stress, thereby influencing purine metabolism and contributing to elevated serum uric acid levels (34, 35). Accordingly, these pathways should be interpreted as biologically plausible but hypothetical, and longitudinal and experimental studies are required to clarify causal relationships.

Finally, several limitations merit consideration. The cross-sectional design precludes causal inference, making it impossible to determine whether elevated LCI precedes the development of hyperuricemia or represents a downstream metabolic consequence. Although we adjusted for a wide range of demographic, occupational, lifestyle, and clinical covariates, residual confounding cannot be excluded, particularly from unmeasured factors such as detailed dietary purine or fructose intake and genetic determinants of uric acid metabolism. In addition, LCI is a derived composite measure, and direct assessment of underlying lipoprotein characteristics—such as particle size, subclass distribution, or remnant lipoprotein concentrations—was unavailable, limiting mechanistic interpretation. Because serum lipids and uric acid were measured at a single visit, intra-individual variability and regression dilution may have occurred, leading to potential non-differential misclassification of exposure and outcome, which would likely bias associations toward the null. Moreover, the study population consisted of oilfield workers from a single region in China, which may restrict the generalizability of the findings to other populations or occupational settings. Not all workers undergoing routine occupational health examinations were included in the analysis, and information on the characteristics of non-participants was not available. As inclusion was based on active employment and attendance at routine examinations, a healthy worker effect cannot be excluded, potentially leading to an underestimation of the true associations. Future studies incorporating prospective designs and more detailed lipidomic and metabolic profiling will be essential to strengthen causal inference and to further elucidate the biological links between LCI and hyperuricemia.

## 5 Conclusion

In conclusion, among normolipidemic oilfield workers, higher LCI was associated with greater odds of hyperuricemia. These results suggest that composite lipid indices may reveal clinically relevant metabolic imbalance that is not captured by conventional lipid panels, and that LCI may serve as a feasible, low-cost adjunct to routine lipid assessment in both clinical and occupational settings. However, its predictive performance, optimal thresholds, and utility for risk stratification require confirmation in future longitudinal studies before clinical implementation.

## Data Availability

The raw data supporting the conclusions of this article will be made available by the authors, without undue reservation.
