# Supplementary material for: Lipoprotein combined index and prevalent hyperuricemia among normolipidemic oilfield workers: a cross-sectional study
Source: Front Endocrinol (Lausanne). 2026 Mar 26;17:1768222. doi: 10.3389/fendo.2026.1768222 (PMC13061720; doi:10.3389/fendo.2026.1768222)
Supplement: Supplementary file 1 [file DataSheet1.pdf]

Supplementary Material

Table S1. Category and definition of covariates.

Table S2. Number and percentage of missing values for each covariate.

Table S3. Baseline characteristics of participants based on hyperuricemia status.

Table S4. Stratified analyses of the association between lipoprotein combined index and hyperuricemia among normolipidemic oilfield workers.

Table S5. Association between lipoprotein combined index and hyperuricemia among normolipidemic oilfield workers, excluding participants with incomplete covariate data.

Table S6. Association between lipoprotein combined index and hyperuricemia among normolipidemic oilfield workers, using the Chinese clinical guideline definition of hyperuricemia.

Table S7. Association between lipoprotein combined index and hyperuricemia among normolipidemic oilfield workers, including participants with extreme lipoprotein combined index values.

Table S8. Association between lipoprotein combined index and hyperuricemia among normolipidemic oilfield workers, categorizing the index into tertiles.

Figure S1. Pearson correlation matrix of lipoprotein combined index, lipid components, body mass index, and serum uric acid.

Table S1. Category and definition of covariates.

Variable	Category and definition
Shift work	Shift work is defined as the regular rotation of individuals to work outside the hours of 8:00 AM to 5:00 PM for a minimum duration of one year.
Chemical substance exposure	Chemical substance exposure is defined as the self-reported exposure of individuals to hazardous chemical substances in the workplace or environment, including benzene, toluene, xylene, hydrogen sulfide, carbon monoxide, nitrogen oxides, carbon tetrachloride, n-hexane, n-pentane, gasoline, etc.
Noise exposure	Noise exposure is defined as the self-reported exposure of individuals to noise levels in the work environment that exceed legal noise standards.
Dust exposure	Dust exposure is defined as the self-reported exposure of individuals to inhalable or respirable dust particles during the work process, which may originate from production processes, material handling, or the surrounding environment.
Cigarette smoking	Cigarette smokers are defined as individuals who smoke at least one cigarette daily for six months or longer.
Alcohol drinking	Alcohol drinking is defined as those who consume alcohol at least once a week and maintain this frequency for six months or longer.
Physical activity	Physical activity is assessed based on questionnaire items asking participants whether they usually engage in moderate-intensity activities — such as slow jogging or moderate-paced cycling—lasting at least 10 minutes per session.
Tea drinking	Tea drinking is defined as consuming tea at least three times per week for a duration of six months or longer.
Salt intake	Salt intake is assessed using a questionnaire item asking participants about their usual daily salt consumption, with response options of ≤6 grams/day and >6 grams/day.
Food diversity	Food diversity is defined by the self-reported number of different food types consumed daily and categorized as <4 types/day and ≥4 types/day.
Body mass index	Body mass index is calculated as weight in kilograms divided by height in meters squared (kg/m^2). Participants were classified into three categories: underweight/normal (<24 kg/m^2), overweight (24–27.9 kg/m^2), and obesity (≥28 kg/m^2).
Estimated glomerular filtration rate	The estimated glomerular filtration rate is calculated to evaluate renal function, which is derived using the Chronic Kidney Disease Epidemiology Collaboration equation.
Hypertension	Hypertension is defined as systolic blood pressure ≥140 mmHg, diastolic blood pressure ≥90 mmHg, self-reported physician diagnosis of hypertension, or current use of antihypertensive medication.
Diabetes	Diabetes is defined as fasting plasma glucose ≥7.0 mmol/L, glycated hemoglobin ≥6.5%, self-reported physician diagnosis of diabetes, or current use of glucose-lowering medication.
Cardiovascular disease	Cardiovascular disease is defined as a self-reported history of coronary heart disease, atherosclerosis, or stroke.

Table S2. Number and percentage of missing values for each covariate.

Covariates	Number of missing values (N)	Missing rate (%)
Age	0	0.00
Sex	0	0.00
Ethnicity	122	6.01
Education level	153	7.54
Marital status	180	8.87
Annual income	226	11.14
Shift work	128	6.31
Chemical substance exposure	109	5.37
Noise exposure	141	6.95
Dust exposure	187	9.22
Cigarette smoking	159	7.84
Alcohol drinking	172	8.48
Tea drinking	109	5.37
Physical activity	119	5.86
Salt intake	242	11.93
Food diversity	134	6.60
Body mass index	2	0.10
Estimated glomerular filtration rate	0	0.00
Hypertension	0	0.00
Diabetes	2	0.10
Cardiovascular disease	2	0.10

Table S3. Baseline characteristics of participants based on hyperuricemia status.

Variables	Total (N = 2,029)	Non-hyperuricemia (N = 1,683)	Hyperuricemia (N = 346)	*P* value
**Age, year**	40.98 (8.32)	41.48 (8.10)	38.53 (8.93)	0.895
**Sex**				<0.001
Male	1088 (53.62)	816 (48.48)	272 (78.61)	
Female	941 (46.38)	867 (51.52)	74 (21.39)	
**Ethnicity**				0.773
Han	1989 (98.03)	1651 (98.10)	338 (97.69)	
Other	40 (1.97)	32 (1.90)	8 (2.31)	
**Education level**				<0.001
High school or below	723 (35.63)	630 (37.43)	93 (26.88)	
College degree	760 (37.46)	635 (37.73)	125 (36.13)	
University graduate or above	546 (26.91)	418 (24.84)	128 (36.99)	
**Marital status**				<0.001
Married	1714 (84.48)	1454 (86.39)	260 (75.14)	
Unmarried/Separated	315 (15.52)	229 (13.61)	86 (24.86)	
**Annual income, thousand (CNY)**				0.001
≤ 100	440 (21.69)	389 (23.11)	51 (14.74)	
101-150	1227 (60.47)	1006 (59.77)	221 (63.87)	
≥ 151	362 (17.84)	288 (17.11)	74 (21.39)	
**Shift work**				0.006
No	588 (28.98)	466 (27.69)	122 (35.26)	
Yes	1441 (71.02)	1217 (72.31)	224 (64.74)	
**Chemical substance exposure**				1.000
No	431 (21.24)	357 (21.21)	74 (21.39)	
Yes	1598 (78.76)	1326 (78.79)	272 (78.61)	
**Noise exposure**				1.000
No	747 (36.82)	620 (36.84)	127 (36.71)	
Yes	1282 (63.18)	1063 (63.16)	219 (63.29)	
**Dust exposure**				0.100
No	1494 (73.63)	1252 (74.39)	242 (69.94)	
Yes	535 (26.37)	431 (25.61)	104 (30.06)	
**Cigarette smoking**				<0.001
No	1287 (63.43)	1118 (66.43)	169 (48.84)	
Yes	742 (36.57)	565 (33.57)	177 (51.16)	
**Alcohol drinking**				<0.001
No	1402 (69.10)	1211 (71.95)	191 (55.20)	
Yes	627 (30.90)	472 (28.05)	155 (44.80)	
**Tea drinking**				0.038
No	1050 (51.75)	889 (52.82)	161 (46.53)	
Yes	979 (48.25)	794 (47.18)	185 (53.47)	
**Physical activity**				0.034

Inactive	467 (23.02)	403 (23.95)	64 (18.50)	
Active	1562 (76.98)	1280 (76.05)	282 (81.50)	
**Salt intake**				0.059
≤ 6 grams/day	1041 (51.31)	880 (52.29)	161 (46.53)	
> 6 grams/day	988 (48.69)	803 (47.71)	185 (53.47)	
**Food diversity**				0.398
< 4 types/day	942 (46.43)	789 (46.88)	153 (44.22)	
≥ 4 types/day	1087 (53.57)	894 (53.12)	193 (55.78)	
**Body mass index, Kg/m^2**				<0.001
Underweight/Normal	1250 (61.61)	1128 (67.02)	122 (35.26)	
Overweight	593 (29.23)	455 (27.04)	138 (39.88)	
Obesity	186 (9.17)	100 (5.94)	86 (24.86)	
**eGFR, mL/min/1.73 m^2**	110.41 (10.27)	110.59 (10.11)	109.56 (10.96)	0.089
**Hypertension**				<0.001
No	1698 (83.69)	1440 (85.56)	258 (74.57)	
Yes	331 (16.31)	243 (14.44)	88 (25.43)	
**Diabetes**				0.565
No	1938 (95.52)	1605 (95.37)	333 (96.24)	
Yes	91 (4.48)	78 (4.63)	13 (3.76)	
**Cardiovascular disease**				0.725
No	1955 (96.35)	1620 (96.26)	335 (96.82)	
Yes	74 (3.65)	63 (3.74)	11 (3.18)	
**Lipoprotein combined index**	11.61 (7.33)	10.89 (6.94)	15.12 (8.16)	<0.001

Table S4. Stratified analyses of the associations between lipoprotein combined index and the risk of hyperuricemia among normolipidemic oilfield workers.

Variable	Quartile 1	Quartile 2	Quartile 3	Quartile 4	*P* for trend
**Age**					
< 40 years	1.00 (Reference)	0.80 (0.43, 1.50), 0.483	1.12 (0.62, 2.07), 0.703	1.38 (0.76, 2.56), 0.294	0.086
≥ 40 years	1.00 (Reference)	1.39 (0.73, 2.71), 0.321	1.65 (0.89, 3.15), 0.120	2.91 (1.62, 5.42), <0.001	<0.001
**Sex**					
Male	1.00 (Reference)	1.27 (0.79, 2.04), 0.325	2.00 (1.27, 3.17), 0.003	1.94 (1.23, 3.08), 0.005	0.002
Female	1.00 (Reference)	0.81 (0.32, 2.04), 0.658	1.14 (0.49, 2.72), 0.757	2.38 (1.08, 5.47), 0.035	0.005
**Shift work**					
No	1.00 (Reference)	1.03 (0.51, 2.12), 0.925	1.03 (0.50, 2.12), 0.940	1.77 (0.90, 3.56), 0.104	0.053
Yes	1.00 (Reference)	0.98 (0.55, 1.77), 0.958	1.88 (1.12, 3.23), 0.020	2.23 (1.33, 3.82), 0.003	<0.001
**Body mass index**					
Underweight/Normal	1.00 (Reference)	0.93 (0.47, 1.82), 0.827	1.47 (0.79, 2.76), 0.225	2.25 (1.24, 4.20), 0.009	0.001
Overweight/Obesity	1.00 (Reference)	1.37 (0.81, 2.35), 0.243	2.18 (1.31, 3.69), 0.003	2.17 (1.30, 3.65), 0.003	0.002

The model was adjusted for age, sex, ethnicity, education level, marital status, annual income, shift work, chemical substance exposure, noise exposure, dust exposure, cigarette smoking, alcohol drinking, tea drinking, physical activity, salt intake, food diversity, body mass index, estimated glomerular filtration rate, hypertension, diabetes, and cardiovascular disease, except for the corresponding stratification variable.

Table S5. Association between lipoprotein combined index and hyperuricemia among normolipidemic oilfield workers, excluding participants with incomplete covariate data.

Variable	Model 1		Model 2		Model 3	
	**OR (95 % CI)**	***P* value**	**OR (95 % CI)**	***P* value**	**OR (95 % CI)**	***P* value**
**Continuous**	1.63 (1.44, 1.86)	<0.001	1.51 (1.31, 1.74)	<0.001	1.26 (1.08, 1.47)	0.004
**Categorical**						
Quartile 1	1.00 (Reference)		1.00 (Reference)		1.00 (Reference)	
Quartile 2	1.45 (0.89, 2.40)	0.138	1.44 (0.86, 2.42)	0.171	1.14 (0.67, 1.94)	0.632
Quartile 3	2.21 (1.40, 3.57)	<0.001	1.92 (1.18, 3.18)	0.010	1.37 (0.82, 2.29)	0.232
Quartile 4	4.14 (2.69, 6.53)	<0.001	3.36 (2.11, 5.49)	<0.001	2.01 (1.22, 3.35)	0.007
P for trend		<0.001		<0.001		<0.001

Model 1, no covariate was adjusted. Model 2, adjusted for age, sex, ethnicity, education level, marital status, and annual income. Model 3, further adjusted for shift work, chemical substance exposure, noise exposure, dust exposure, cigarette smoking, alcohol drinking, tea drinking, physical activity, salt intake, food diversity, body mass index, estimated glomerular filtration rate, hypertension, diabetes, and cardiovascular disease. OR, odds ratio; CI, confidence interval.

Table S6. Association between lipoprotein combined index and hyperuricemia among normolipidemic oilfield workers, using the Chinese clinical guideline definition of hyperuricemia.

Variable	Model 1		Model 2		Model 3	
	OR (95 % CI)	*P* value	OR (95 % CI)	*P* value	OR (95 % CI)	*P* value
**Continuous**	1.91 (1.69, 2.16)	<0.001	1.63 (1.43, 1.87)	<0.001	1.36 (1.19, 1.56)	<0.001
**Categorical**						
Quartile 1	1.00 (Reference)		1.00 (Reference)		1.00 (Reference)	
Quartile 2	1.36 (0.86, 2.17)	0.196	1.26 (0.78, 2.04)	0.352	1.01 (0.63, 1.63)	0.962
Quartile 3	2.65 (1.75, 4.09)	<0.001	1.96 (1.27, 3.07)	0.003	1.46 (0.95, 2.28)	0.091
Quartile 4	5.01 (3.39, 7.58)	<0.001	3.36 (2.22, 5.19)	<0.001	2.08 (1.37, 3.21)	<0.001
P for trend		<0.001		<0.001		<0.001

Model 1, no covariate was adjusted. Model 2, adjusted for age, sex, ethnicity, education level, marital status, and annual income. Model 3, further adjusted for shift work, chemical substance exposure, noise exposure, dust exposure, cigarette smoking, alcohol drinking, tea drinking, physical activity, salt intake, food diversity, body mass index, estimated glomerular filtration rate, hypertension, diabetes, and cardiovascular disease. OR, odds ratio; CI, confidence interval.

Table S7. Association between lipoprotein combined index and hyperuricemia among normolipidemic oilfield workers, including participants with extreme lipoprotein combined index values.

Variable	Model 1		Model 2		Model 3	
	**OR (95 % CI)**	***P* value**	**OR (95 % CI)**	***P* value**	**OR (95 % CI)**	***P* value**
**Continuous**	1.65 (1.49, 1.84)	<0.001	1.55 (1.38, 1.75)	<0.001	1.33 (1.17, 1.51)	<0.001
**Categorical**						
Quartile 1	1.00 (Reference)		1.00 (Reference)		1.00 (Reference)	
Quartile 2	1.38 (0.93, 2.06)	0.111	1.39 (0.92, 2.12)	0.117	1.12 (0.73, 1.73)	0.607
Quartile 3	2.04 (1.41, 2.98)	<0.001	1.82 (1.23, 2.73)	0.003	1.37 (0.91, 2.09)	0.136
Quartile 4	4.08 (2.89, 5.85)	<0.001	3.46 (2.38, 5.12)	<0.001	2.22 (1.49, 3.35)	<0.001
P for trend		<0.001		<0.001		<0.001

Model 1, no covariate was adjusted. Model 2, adjusted for age, sex, ethnicity, education level, marital status, and annual income. Model 3, further adjusted for shift work, chemical substance exposure, noise exposure, dust exposure, cigarette smoking, alcohol drinking, tea drinking, physical activity, salt intake, food diversity, body mass index, estimated glomerular filtration rate, hypertension, diabetes, and cardiovascular disease. OR, odds ratio; CI, confidence interval.

Table S8. Association between lipoprotein combined index and hyperuricemia among normolipidemic oilfield workers, categorizing the index into tertiles.

Variable	Model 1		Model 2		Model 3	
	OR (95 % CI)	*P* value	OR (95 % CI)	*P* value	OR (95 % CI)	*P* value
**Continuous**	1.76 (1.56, 1.98)	<0.001	1.63 (1.43, 1.85)	<0.001	1.37 (1.20, 1.58)	<0.001
**Categorical**						
Tertile 1	1.00 (Reference)		1.00 (Reference)		1.00 (Reference)	
Tertile 2	1.66 (1.19, 2.34)	0.003	1.61 (1.13, 2.32)	0.009	1.27 (0.87, 1.85)	0.216
Tertile 3	3.65 (2.69, 5.01)	<0.001	3.15 (2.26, 4.45)	<0.001	2.15 (1.51, 3.09)	<0.001
P for trend		<0.001		<0.001		<0.001

Model 1, no covariate was adjusted. Model 2, adjusted for age, sex, ethnicity, education level, marital status, and annual income. Model 3, further adjusted for shift work, chemical substance exposure, noise exposure, dust exposure, cigarette smoking, alcohol drinking, tea drinking, physical activity, salt intake, food diversity, body mass index, estimated glomerular filtration rate, hypertension, diabetes, and cardiovascular disease. OR, odds ratio; CI, confidence interval.

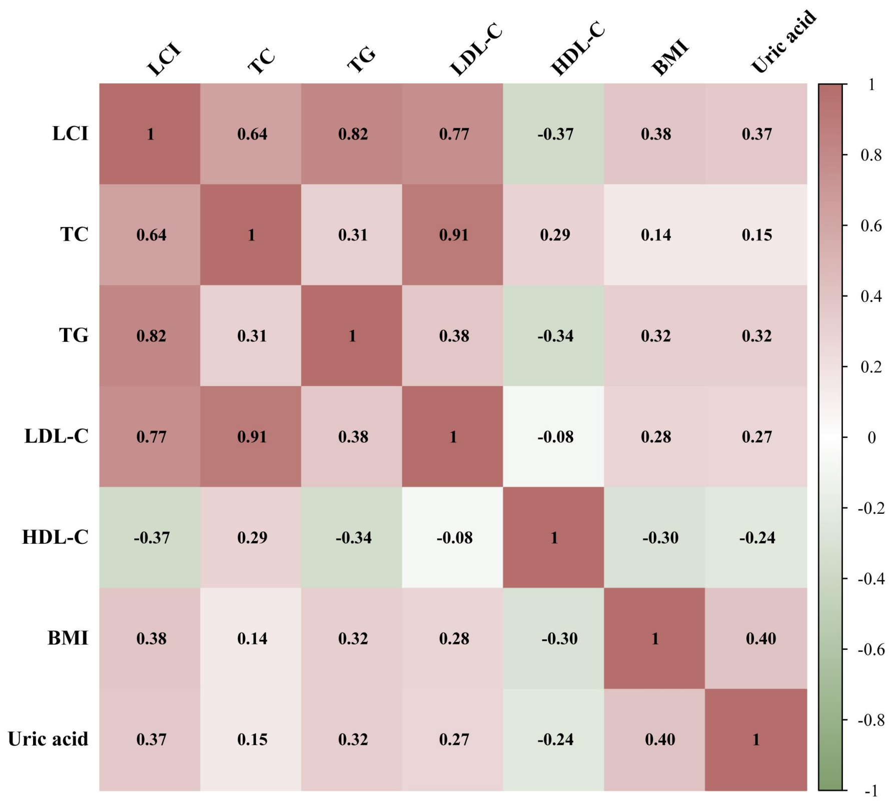

Figure S1. Pearson correlation matrix of lipoprotein combined index, lipid components, body mass index, and serum uric acid. Pearson correlation coefficients were calculated to assess pairwise associations between lipoprotein combined index, total cholesterol, triglycerides, low-density lipoprotein cholesterol, higher high-density lipoprotein cholesterol, body mass index, and serum uric acid. Color intensity represents the strength and direction of correlations. LCI, lipoprotein combined index; TC, total cholesterol; TG, triglycerides; LDL-C, low-density lipoprotein cholesterol; HDL-C, higher high-density lipoprotein cholesterol; BMI, body mass index.
